# Association between three autoimmune diseases: vitiligo, primary biliary cirrhosis, and Sjögren's syndrome^?^^??^

**DOI:** 10.1016/j.abd.2018.07.002

**Published:** 2019-10-23

**Authors:** Lara Silveira Abdo-Aguiar, Caio César Silva de Castro

**Affiliations:** a Dermatology Service, Hospital Santa Casa de Misericórdia de Curitiba, Curitiba, PR, Brazil; b Discipline of Dermatology, Pontifícia Universidade Católica do Paraná, Curitiba, PR, Brazil

**Keywords:** Autoimmunity, Liver cirrhosis, biliary, Sjogren's syndrome, Vitiligo

## Abstract

Although the association of multiple autoimmune diseases has already been widely described, no reports of the association between vitiligo, primary biliary cirrhosis and Sjogren's syndrome were retrieved in the SciELO and PubMed databases. The authors describe the case of a female patient who was diagnosed with primary biliary cirrhosis and Sjogren's syndrome at age 54. At age 58, she developed vitiligo restricted to the face, associated with significant impairment of self-esteem and quality of life. Antinuclear antibody was negative at the onset of the condition, but became positive after phototherapy initiation. In general, the occurrence of multiple autoimmune diseases in the same patient is known as a mosaic of autoimmunity. However, specific mechanisms appear to interconnect primary biliary cirrhosis and Sjogren's syndrome, such as PDC-E2-mediated generalized epithelitis.

## Introduction

Vitiligo is a chronic autoimmune disease characterized by the appearance of hypochromic and achromic macules and patches on the skin and mucous membranes, due to the disappearance of melanocytes in the affected area.^1^ In turn, primary biliary cirrhosis (PBC) is a chronic hepatic autoimmune disease characterized by the destruction of the epithelial cell lining the intrahepatic bile ducts, progressively evolving to fibrosis and cirrhosis.^2^ Sjogren's syndrome (SS) is a chronic autoimmune condition affecting exocrine glands, especially the lacrimal and salivary glands. The main clinical manifestations are xerostomia and xerophthalmia. The primary form of the disease is more prevalent in females than males, in a ratio of 9:1, affecting approximately 0.5% of the population, a prevalence similar to that of systemic lupus erythematosus and systemic sclerosis.^3^ It is well known that the occurrence of an autoimmune disease increases the risk of other autoimmune diseases in the same patient, a phenomenon that has been explored in previously published reports and case series; however, the association between common vitiligo, PBC and SS has not been published to date.^4^

## Case report

**Figure 1:**
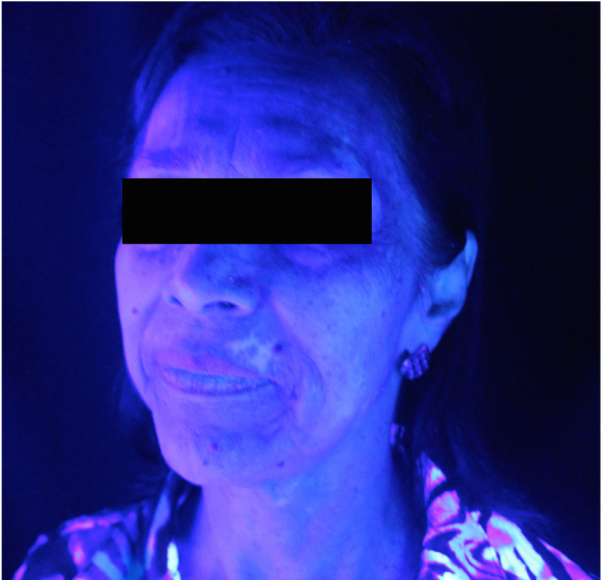
Vitiligo on the face, front position (Wood's light examination).

**Figure 2:**
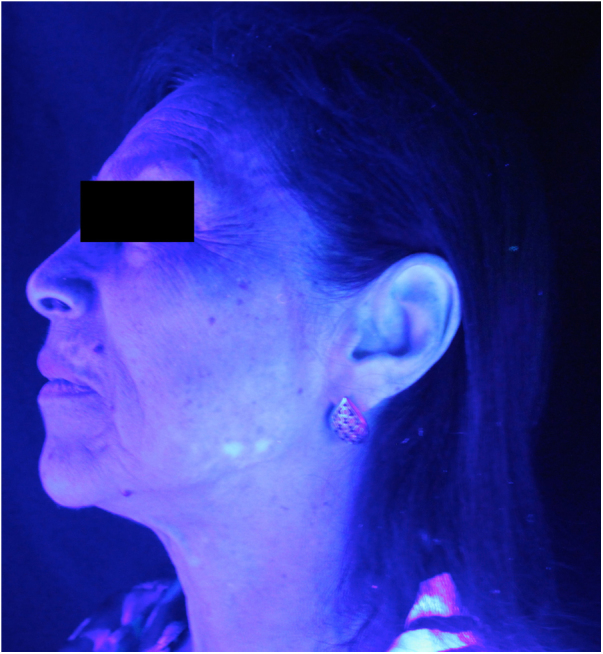
Vitiligo on left hemiface (Wood's light examination).

A female patient was diagnosed with PBC and SS at 54 years of age. She started treatment with ursodeoxycholic acid, deflazacort, hydroxychloroquine, and pilocarpine, reaching satisfactory disease control. Subsequently, at age 58, she evolved with hypochromic and, later, achromic lesions characteristic of vitiligo restricted to the facial area (Figure 1, Figure 2), associated with significant impairment in self-esteem and quality of life. She had a positive family history of vitiligo in a third-degree relative. At the first evaluation, the patient had negative antinuclear antibody (ANA) and anemia due to chronic iron deficiency, requiring blood transfusion. For vitiligo, topical treatment with tacrolimus and phototherapy with narrow band UVB were indicated, which stabilized the progression of the disease, without new lesions appearing on the face or other areas of the skin. After starting phototherapy, laboratory tests showed positive ANA with mixed pattern – thick reticulated speckled 1:320 and fine speckled 1:640 – with no other changes.

## Discussion

Autoimmune diseases are chronic conditions that result from the loss of immune tolerance to self-antigens. The origin of autoimmunity mechanisms remains partially known, and a combination of genetic, immunological, environmental, and hormonal factors is linked to its development. Autoimmune diseases can be classified as either organ-specific (*e.g.*, myasthenia gravis, Graves’ disease, and polymyositis) or multisystem diseases (*e.g.*, systemic lupus erythematosus, rheumatoid arthritis, and systemic sclerosis).^2^

Vitiligo is a cutaneous disease of chronic and autoimmune nature that, although occurring sporadically, is known to have a certain degree of heredity. Studies have previously demonstrated the involvement of different genes in its pathogenesis, but to date there is no known evidence of a validated serum or tissue marker associated with its occurrence. Case reports and series have associated vitiligo with other autoimmune diseases, and studies have reported common genes between this condition and systemic lupus erythematosus and Hashimoto's thyroiditis.^1^

PBC is considered a prototype of autoimmune disease due to the characteristic antimitochondrial autoantibody, its homogeneous clinical presentation, and the specificity of the anatomopathological findings. Genetic deficiencies and mechanisms of immune regulation take part in the pathogenesis of the disease, which results in damage exclusively to the small- and medium-caliber bile duct cells; over time, consequently and progressively, a state of chronic autoimmune cholangitis develops.^4^, ^5^

In 2012, Efe et al. carried out a multicenter study of 71 patients with PBC and autoimmune hepatitis (AIH) to analyze the association of these with other autoimmune diseases. The data indicated that 76.1% of the patients had a positive anti-nuclear factor, 74.6% had antimitochondrial antibodies, and 52.1% had both markers. In 31 patients (43.6%), 14 extrahepatic autoimmune comorbidities were identified, with predominance for thyroid disorders in 13 participants of the study. Other findings included SS in six participants (8.4%) and psoriasis, celiac disease, and rheumatoid arthritis in three patients each (4.2%). Vitiligo was observed in two patients (2.8%); the same proportion was observed for systemic lupus erythematosus.^2^

The concomitant occurrence of multiple autoimmune diseases in the same patient draws attention to the presence of common mechanisms. The concept of mosaic of autoimmunity has been proposed to describe this condition and demonstrated in reports and series of cases, as mentioned above, although the specific immunological and genetic mechanisms have not yet been fully elucidated.^2^

Patients with autoimmune hepatic diseases, such as PBC and AIH, may also present with other organ-specific or multisystemic autoimmune conditions. In the aforementioned individuals, SS was the most frequent multisystem autoimmune comorbidity.^2^ Two previously published studies analyzed 34 SS patients who had a concomitant elevation of hepatic enzymes, among which a diagnosis of PBC or AIH was made in 15 and nine patients, respectively.^6^, ^7^

A significant portion of patients with PBC suffer from the so-called sicca syndrome, and some of them present classical SS. Both PBC and SS are characterized by inflammation and immune-mediated destruction of epithelial tissue; moreover, the PDC-E2 target antigen has been identified in both bile duct and salivary gland epithelium, which strongly demonstrates the association between both conditions.^2^, ^5^ In addition, the finding that the salivary and lacrimal glands, as well as the urinary tract epithelium, may also be damaged in PBC has led to the hypothesis that PBC, similarly to SS, can be considered a generalized epithelitis.^8^ To date, there is no evidence that vitiligo has the same pathophysiological mechanism.

Finally, the association between this patient's comorbidities and their underlying pathophysiological mechanism was clarified; the triple association was explained by the mosaic of autoimmunity and the occurrence of epithelitis.

## Financial support

None declared.

## Author's contributions

Lara Silveira Abdo Aguiar: Elaboration and writing of the manuscript; obtaining, analyzing and interpreting the data; intellectual participation in propaedeutic and/or therapeutic conduct of the cases studied.

Caio César Silva de Castro: Conception and planning of the study; effective participation in research orientation; intellectual participation in propaedeutic and/or therapeutic conduct of the cases studied; critical review of the literature.

## Conflicts of interest

None declared.
